# New Recurrent Structural Aberrations in the Genome of Chronic Lymphocytic Leukemia Based on Exome-Sequencing Data

**DOI:** 10.3389/fgene.2019.00854

**Published:** 2019-09-20

**Authors:** Adrián Mosquera Orgueira, Beatriz Antelo Rodríguez, José Ángel Díaz Arias, Marta Sonia González Pérez, José Luis Bello López

**Affiliations:** ^1^Research Group on Lymphoproliferative Diseases, Health Research Institute of Santiago de Compostela (IDIS), Santiago de Compostela, Spain; ^2^Complexo Hospitalario Universitario de Santiago de Compostela (CHUS), Division of Hematology, SERGAS, Santiago de Compostela, Spain; ^3^Department of Medicine, University of Santiago de Compostela, Santiago de Compostela, Spain

**Keywords:** copy number aberration, chronic lymphocytic leukemia, driver, time to treatment, overall survival

## Abstract

Chronic lymphocytic leukemia (CLL) is the most frequent lymphoproliferative syndrome in Western countries, and it is characterized by recurrent large genomic rearrangements. During the last decades, array techniques have expanded our knowledge about CLL’s karyotypic aberrations. The advent of large sequencing databases expanded our knowledge cancer genomics to an unprecedented resolution and enabled the detection of small-scale structural aberrations in the cancer genome. In this study, we have performed exome-sequencing-based copy number aberration (CNA) and loss of heterozygosity (LOH) analysis in order to detect new recurrent structural aberrations. We describe 54 recurrent focal CNAs enriched in cancer-related pathways, and their association with gene expression and clinical evolution. Furthermore, we discovered recurrent large copy number neutral LOH events affecting key driver genes, and we recapitulate most of the large CNAs that characterize the CLL genome. These results provide “proof-of-concept” evidence supporting the existence of new genes involved in the pathogenesis of CLL.

## Introduction

Chronic lymphocytic leukemia (CLL) is the most frequent lymphoproliferative disease in Western populations, and it is characterized by its clinical and genetic heterogeneity. Döhner et al. (2000) described the widely used cytogenetic classification of CLL based on the most prevalent chromosomal aberrations in the CLL genome (Döhner et al., 2000), that is, trisomy 12 and deletions in 13q14.2–14.3, 11q22.3, and 17p13.1. Since that moment, new genome-wide studies have revealed new recurrent genomic aberrations, such as trisomy 19, amplifications at 2p and 8q, and deletions at 8p, 6q21, 18p, and 20p (Pfeifer et al., 2007; Landau et al., 2015). Similarly, a wealth of genomic and epigenomic modulators of CLL’s clinical aggressivity have been discovered (Nadeu et al., 2018), such as point mutations in *NOTCH1, SF3B1, ATM, TP53*, and *POT1* and the absence of somatic hypermutation at the *IGHV* locus. It has been observed that copy number aberrations (CNAs) in CLL genomes tend to be acquired early in disease evolution and usually remain stable, whereas the mutational heterogeneity can increase (Nadeu et al., 2018). Indeed, mounting evidence indicates that the accumulation of these cytogenomic events modulates CLL proliferation and clinical aggressivity to a great extent (Raponi et al., 2018; Gruber et al., 2019), acting as drivers of genomic complexity and clonal evolution (Edelmann et al., 2017; Yu et al., 2017; Hernández-Sánchez et al., 2019) and accumulating in relapsed cases (Ljungström et al., 2016; Leeksma et al., 2019).

CNAs and copy number neutral loss of heterozygosity (CNN-LOH) are oncogenic mechanisms that induce gene-dosage effects, disrupt coding sequences, cause structural rearrangements, or potentiate epigenetic effects. Oncogenes are frequently affected by copy number gains, while tumor suppressor genes tend to be deleted. Massive array-based techniques such as array comparative genomic hybridization and single-nucleotide polymorphism (SNP) arrays have enabled the analysis of structural aberrations on cancer genomes to an unprecedented resolution of 10–100 kb. With the development of massive sequencing technologies, large databases of cancer sequence data have been published. This motivated the development of a variety of CNA detection algorithms from exome-sequencing data, which have the additional benefit of detecting smaller CNA events at the expense of increased false discoveries and reduced sensitivity and specificity (Nam et al., 2016). These methods are specifically designed to face particular issues, particularly those inherent to the sequencing protocol (such as biases induced by hybridization, GC content, and read mappability), due to cancer biology (ploidy estimation and subclonality) (Zare et al., 2017) and due to the presence or absence of matched controls (Kim et al., 2017).

In this analysis, we used previously published exome-seq data in order to detect small recurrent structural events involved in the pathogenesis of CLL. Our results not only reproduce the known cytogenetic aberrations in CLL but also support the existence of multiple recurrent focal CNAs and CNN-LOH affecting key oncogenic pathways, some of which are clearly associated with higher proliferative capacity, shorter survival, and altered gene expression. We conclude that focal CNAs may be more relevant than previously expected in the pathogenesis of CLL, and they merit further consideration for prognostic stratification.

## Methodology

### Data Source

The *International Cancer Genome Consortium* (ICGC) Data Access Committee granted us access to the CLL sequencing data (Ramsay et al., 2013) deposited in the *European Genome-Phenome Database* (EGA) under *DACO-1040945*. For this analysis, we used exome-seq data from matched control and tumor samples of CLL cases under the accession code *EGAD00001001464*. Patient characteristics can be consulted in **Table 1**.

**Table 1:** Patient characteristics.

Patient characteristics	
Number of cases	441
Males/females	59.7%/40.3%
Median age at diagnosis	62.5 years
% of MBL	11.20%
% of Binet A	77.12%
% of Binet B	9.61%
% of Binet C	2.06%
% unmutated IGHV	35.03%

MBL, monoclonal B-cell lymphocytosis.

### Data Preprocessing

Samples were processed by Puente et al. (2015) as described in their original paper (Puente et al., 2015). Briefly, 3 μg of genomic DNA was used for paired-end sequencing library construction, followed by enrichment in exomic sequences using the *SureSelect Human All Exon 50Mb* kit (Agilent Technologies). Next, DNA was pulled down using magnetic beads with streptavidin, followed by 18 cycles of amplification. Sequencing was performed on an Illumina GAIIx or on a HiSeq2000 sequencer (2 × 76 bp). Exome-seq data were aligned to the reference genome (GRCh37.75) using *bwa* (Li and Durbin, 2009). Duplicate read removal, sorting, and indexing were done using *samtools* (Li et al., 2009). Base quality score recalibration was made with *BamUtil* (Breese and Liu, 2013) using a logistic regression model.

### CNA and CNN-LOH Detection

We analyzed paired tumor-normal exome-sequencing data with Control-FREEC version 11.3 in order to identify somatic CNA and CNN-LOH regions (Boeva et al., 2012). Control-FREEC uses aligned reads to construct and normalize a copy number profile and a B-allele frequency (BAF) profile. Then, it performs profile segmentation and infers genotype status for each segment using both copy number and allelic frequency information. Finally, genomic aberrations are identified and annotated.

The following specifications were used: “window = 0,” “ploidy = 2,” “breakPointThreshold = 1.2,” “noisyData = TRUE,” “readCountThreshold = 50,” “forceGContentNormalization = 1,” “contaminationAdjustment = TRUE,” “telocentromeric = 50000,” and “mateOrientation = 0.” BAFs were estimated using all variants reported in dbSNP (version 150) with a minimal coverage per variant position of 5 reads and a minimal sequencing quality per position of 20 Phred. Variant calling was limited to regions covered in the SureSelect Exome Capture 50Mb version 4 kit.

### LOH and CNA Selection and Filtering

CNN-LOH were called with p-values < 0.05 (Kolmogorov–Smirnov test) and an uncertainty upper threshold of 5%. Regions with lowmappability according to UCSC 75-bp mappability tracks (score below 0.5) were filtered out. As expected, we observed regions that seemed prone to CNA erroneous detection. Thus, we decided to apply a hard filter and discard those CNAs significantly enriched in both amplifications and deletions, as well as those located near a telomeric or centromeric region. CNA events were detected using GISTIC2.0 (Beroukhim et al., 2007). GITIC2.0 was run with default parameters plus the arm peel-off filter. Focal recurrent CNAs were defined as those spanning less than 50% of a chromosome arm with a residual *q*-value < 0.05 and a wide peak size below 10 megabases. A 1 − log2 tumor/normal ratio above 0.3 was used to define amplifications, and a ratio below −0.3 was used to define deletions.

### Survival Analysis

Variables associated with time to treatment (TTT) and overall survival (OS) were analyzed using Cox regression as implemented in the *survival* R package (Therneau and Grambsch, 2000; R Development Core Team, 2011; Therneau, 2015). Assessment of the proportional hazards assumption was performed using the *cox.zph* function. We created two different models: a univariate model that only includes CNA status for each gene and an adjusted model that included variables associated with clinical outcome at a *p*-value < 0.2 in a univariate model. The combined model (CM) for TTT analysis included as covariates donor sex, stage at diagnosis (monoclonal B-cell lymphocytosis (MBL), Binet A, Binet B, and Binet C), and *IGHV* mutational status, while the CM for OS analysis included stage at diagnosis, *IGHV* mutation status, and age at diagnosis. The Benjamini–Hochberg (BH) method was used to adjust for multiple testing.

### RNAseq Analysis and Correlation With CNA Status

Two hundred twenty patients had matched RNAseq data of CLL-purified cells (accession IDs *EGAD00001001443* and *EGAD00001000258*). Illumina adapters were removed using *cutadapt* (Martin, 2011), and alignment to the human reference genome (GRCh37) was performed using *Hisat2* (Kim et al., 2015) with default specifications. We used the *Hisat2*-provided Hierarchical Graph FM index for GRCh37 with SNP and Ensembl transcript information. Bam files were sorted and indexed using *samtools* (Li et al., 2009). Bam files were processed in *R* (R Development Core Team, 2011) according to the RNAseq gene expression protocol developed by Love et al. (2015). Briefly, bam files were read using *Rsamtools* (Morgan et al., 2017), followed by gene-level expression estimation using the *SummarizeOverlaps* function from the *GenomicAlignments* package (Lawrence et al., 2013). Gene models in GTF format were downloaded from Ensembl (GRCh37.75 version) (Yates et al., 2016). A log2-transformation on normalized frames per kilobase counts was performed. Focal CNAs were classified according to Gistic into low-range events (tumor/normal log2 ratio > 0.3 and <0.9 for amplifications and less than −0.9 and more than −0.3 for deletions) and higher-range events (tumor/normal log2 ratio > 0.9 for amplifications and less than −0.9 for deletions). Correlation between CNA status and gene expression was performed using Spearman’s correlation. A minimum of 5 CNA events with matched transcriptomic data was set for analysis. Furthermore, immunoglobulin and T-cell receptor gene rearrangements were not included. *p*-values were adjusted for multiple testing using the BH method.

## Results

### Focal CNA Regions and Their Association With TTT and OS

**Figure 1:**
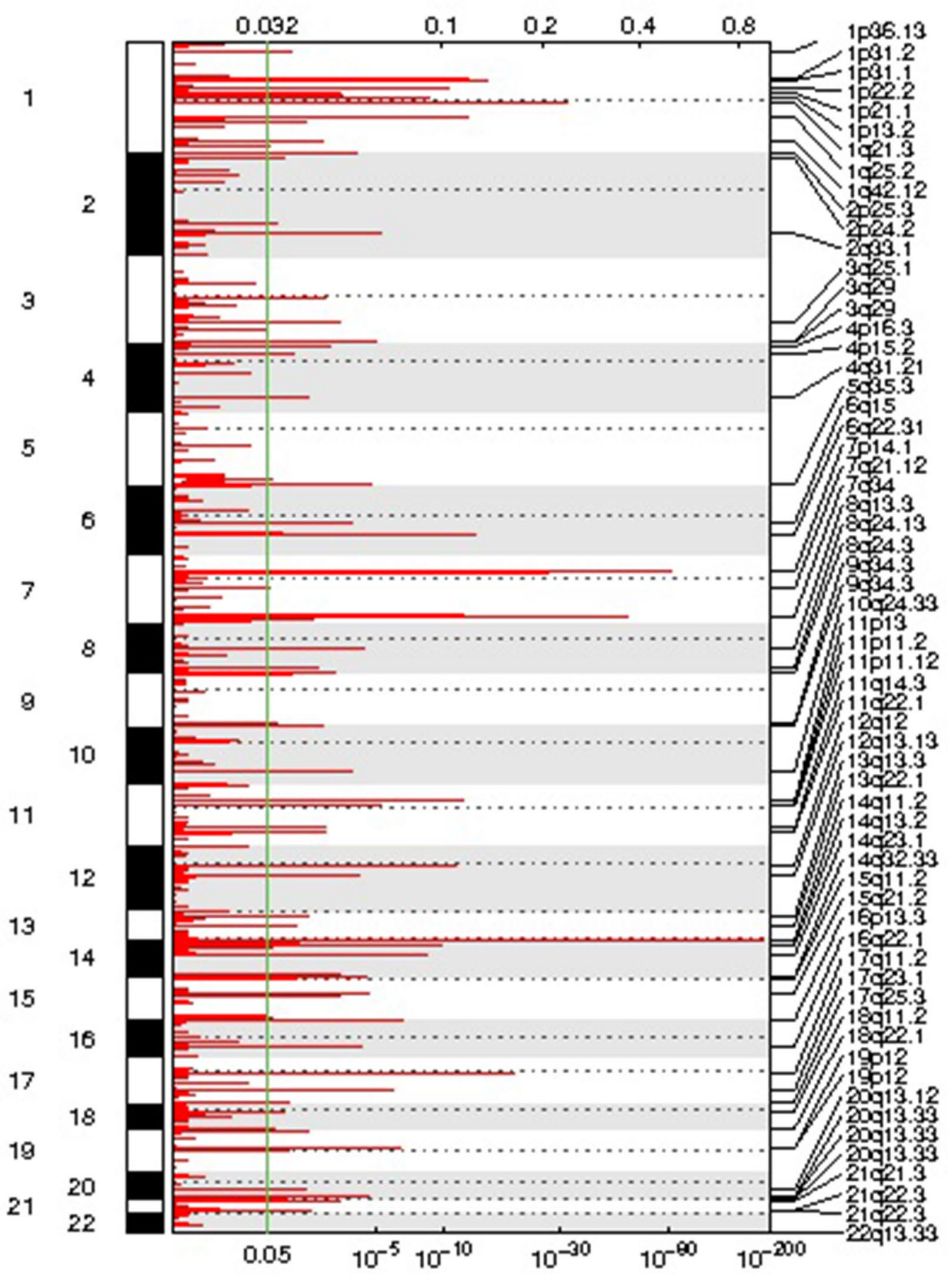
Representation of the focal amplifications detected in the chronic lymphocytic leukemia (CLL) genomes along with their corresponding statistical significance.

**Figure 2:**
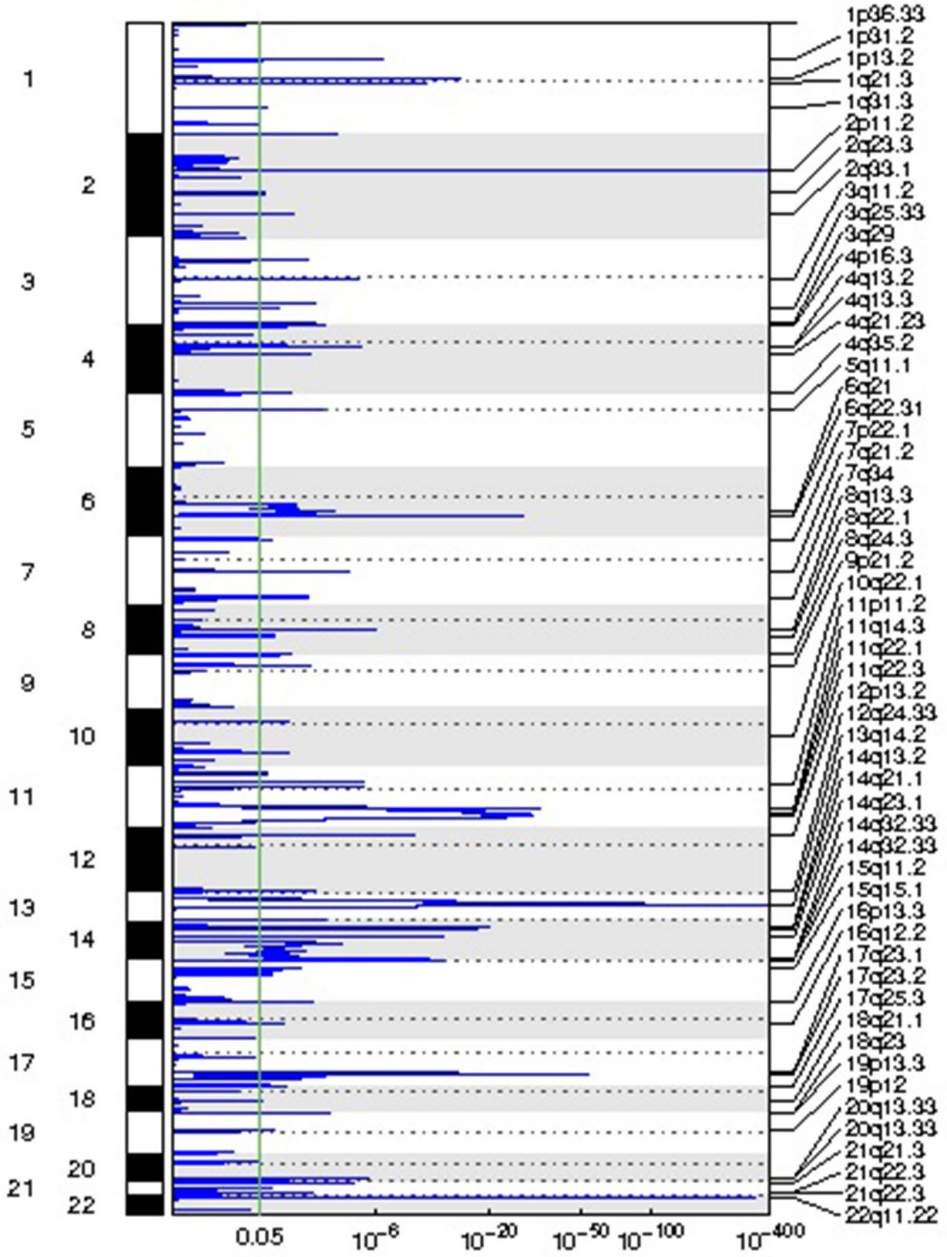
Representation of the focal deletions detected in the chronic lymphocytic leukemia (CLL) genomes along with their corresponding statistical significance.

We identified 54 recurrent focal CNAs in the CLL genome (residual *q*-value < 0.05, **Supplemental Figure 1**, **Table 2**). Among them, there were 31 recurrent amplifications with a wide peak size of 75.1 kb (**Figure 1**) and 23 recurrent deletions with a median wide peak size of 405 kb (**Figure 2**).

**Table 2:** Recurrent focal amplifications and deletions in the chronic lymphocytic leukemia (CLL) genome, including their frequency, wide peak region, length, q-value, residual *q*-value, and involved genes.

Unique Name	Cases	Cytoband	Wide Peak Boundaries	Length	Q value	Residual Q value	Genes in Wide Peak
Amplification Peak 3	9	1p31.1	chr1:74500015–74621552	1.22E+05	4.80E−16	1.86E−11	*LRRIQ3, FPGT, TNNI3K*
Amplification Peak 4	9	1p22.2	chr1:91784576–91813183	2.86E+04	3.13E−11	5.32E−09	*HFM1*
Amplification Peak 8	11	1q25.2	chr1:176093431–176153873	6.04E+04	2.73E−13	8.89E−06	*RFWD2*
Amplification Peak 9	3	1q42.12	chr1:225152123–225211696	59,573	1.71E−03	1.71E−03	*DNAH14*
Amplification Peak 14	15	3q25.1	chr3:149563840–149684409	120,569	4.78E−04	1.92E−03	*PFN2, RNF13*
Amplification Peak 15	14	3q29	chr3:195053676–195063318	9.64E+03	1.01E−05	1.72E−05	*ACAP2*
Amplification Peak 17	6	4p16.3	chr4:3449609–3495293	45,684	9.11E−04	9.11E−04	*HGFAC, DOK7*
Amplification Peak 18	5	4p15.2	chr4:26622159–26641959	19,800	0.01	0.01	*TBC1D19*
Amplification Peak 19	5	4q31.21	chr4:145658875–146010087	351,212	5.17E−03	5.17E−03	*ANAPC10, HHIP*
Amplification Peak 20	10	5q35.3	chr5:180052785–180167076	1.14E+05	1.68E−05	1.68E−05	*FLT4, OR2Y1*
Amplification Peak 22	4	6q15	chr6:89479426–89563571	84,145	1.47E−04	1.47E−04	*RNGTT*
Amplification Peak 24	70	7p14.1	chr7:38284691–38357086	7.24E+04	1.62E−82	1.62E−82	*TCRG locus*
Amplification Peak 28	19	8q24.13	chr8:124810460–124812196	1,736	2.36E−03	0.02	*FAM91A1*
Amplification Peak 30	6	9q34.3	chr9:138905634–139092618	186,984	0.03	0.03	*LHX3, C9orf69, NACC2*
Amplification Peak 31	8	9q34.3	chr9:140161390–140246050	84,660	1.71E−03	1.71E−03	*COBRA1, C9orf167, EXD3, NRARP*
Amplification Peak 32	6	10q24.33	chr10:105073962–105140579	66,617	1.28E−04	1.28E−04	*TAF5, PCGF6*
Amplification Peak 33	26	11p13	chr11:32676388–32705141	2.88E+04	7.50E−13	5.84E−12	*CCDC73, WT1*
Amplification Peak 34	2	11p11.2	chr11:45935717–45955761	20,044	0.04	0.04	*PEX16, PHF21A, GYLTL1B*
Amplification Peak 40	11	13q13.3	chr13:35622613–35697715	75,102	5.17E−03	5.17E−03	*NBEA*
Amplification Peak 41	4	13q22.1	chr13:73530979–73643032	112,053	0.01	0.01	*KLF5, PIBF1*
Amplification Peak 42	188	14q11.2	chr14:22749319–22925867	1.77E+05	4.44E−186	2.84E−185	*TCRA locus*
Amplification Peak 45	12	14q32.33	chr14:105059814–105172511	1.13E+05	2.70E−05	4.01E−04	*INF2, TMEM179, MIR4710, AKT1*
Amplification Peak 50	8	16q22.1	chr16:66819728–66824959	5.23E+03	5.54E−05	5.54E−05	*CCDC79, NAE1*
Amplification Peak 51	12	17q11.2	chr17:30228618–30321772	9.32E+04	2.72E−20	2.23E−17	*SUZ12, UTP6*
Amplification Peak 53	6	17q25.3	chr17:79389937–79430022	40,085	0.02	0.02	*hsa-mir-3186, BAHCC1, MIR3186*
Amplification Peak 54	7	18q11.2	chr18:21229308–21329567	100,259	0.02	0.02	*LAMA3, ANKRD29*
Amplification Peak 55	15	18q22.1	chr18:66365105–66377388	12,283	0.03	0.03	*TMX3*
Amplification Peak 57	8	19p12	chr19:20221949–20317969	96,020	0.02	0.03	*ZNF90, ZNF486*
Amplification Peak 59	3	20q13.12	chr20:42694274–42813092	118,818	5.94E−03	5.94E−03	*JPH2, TOX2*
Amplification Peak 62	5	20q13.33	chr20:62705116–62831313	126,197	0.02	0.02	*NPBWR2, MYT1, OPRL1, RGS19, C20orf201*
Amplification Peak 66	4	22q13.33	chr22:50967459–51008050	40,591	1.39E−03	1.39E−03	*CPT1B, TYMP, KLHDC7B, CHKB-CPT1B, ODF3B, SYCE3*
Deletion Peak 7	193	2p11.2	chr2:88894337–95722218	6,827,881	0	0	*IGK locus*
Deletion Peak 8	4	2q23.3	chr2:152234651–152663572	428,921	0.04	0.04	*NEB, RIF1*
Deletion Peak 13	12	3q25.33	chr3:159713418–160118695	405,277	0.02	0.04	*IFT80, C3orf80, IL12A*
Deletion Peak 16	19	4q13.2	chr4:69344747–69691521	3.47E+05	7.11E−06	3.92E−05	*UGT2B15, UGT2B17*
Deletion Peak 17	8	4q13.3	chr4:70897589–71020148	122,559	0.02	0.02	*HTN1, CSN1S2AP, CSN1S2BP*
Deletion Peak 18	9	4q21.23	chr4:84367119–84457735	90,616	1.71E−03	6.90E−03	*MRPS18C, FAM175A*
Deletion Peak 21	15	6q21	chr6:110797012–111583801	786,789	1.74E−04	1.74E−04	*AMD1, CDK19, RPF2, GTF3C6, SLC16A10, GSTM2P1*
Deletion Peak 23	12	7p22.1	chr7:5269419–5920366	650,947	0.02	0.02	*hsa-mir-589, ACTB, FSCN1, RNF216, FBXL18, TNRC18, SLC29A4, ZNF815*
Deletion Peak 24	11	7q21.2	chr7:92147625–92247368	9.97E+04	3.11E−05	9.98E−05	*RBM48, MGC16142, FAM133B, LOC728066, CDK6*
Deletion Peak 27	11	8q22.1	chr8:95690341–95840099	149,758	0.02	0.02	*DPY19L4, ESRP1, TP53INP1*
Deletion Peak 29	3	9p21.2	chr9:26116211–27109465	993,254	1.62E−03	1.62E−03	*PLAA, LRRC19, C9orf82, IFT74, TEK*
Deletion Peak 32	33	11q14.3	chr11:89185111–89443615	2.59E+05	1.36E−34	1.06E−22	*FOLH1B, NOX4*
Deletion Peak 34	44	11q22.3	chr11:110018375–110449975	4.32E+05	1.18E−31	1.13E−18	*FDX1, RDX, ATM*
Deletion Peak 37	12	12q24.33	chr12:132335406–132436802	101,396	1.09E−03	1.05E−03	*ULK1, PUS1*
Deletion Peak 38	200	13q14.2	chr13:50306479–51501752	1,195,273	0	0	*hsa-mir-15a, DLEU2, TRIM13, DLEU1, SPRYD7, ST13P4, DLEU7, CTAGE10P, KCNRG, MIR15A, MIR16-1, MIR3613*
Deletion Peak 40	41	14q21.1	chr14:39721874–39871082	1.49E+05	1.56E−18	3.83E−10	*CTAGE5, LOC100288846, MIA2, FBXO33*
Deletion Peak 42	27	14q32.33	chr14:104506663–105201322	6.95E+05	1.61E−11	1.05E−03	*hsa-mir-203, KIF26A, INF2, ASPG, TMEM179, C14orf180, MIR203, MIR3545, MIR4710*
Deletion Peak 43	33	14q32.33	chr14:105861125–107349540	1.49E+06	1.89E−13	6.44E−08	*CRIP1, CRIP2, ELK2AP, ADAM6, MTA1, KIAA0125, TMEM121, C14orf80, LINC00226, LINC00221, TEX22*
Deletion Peak 46	10	15q15.1	chr15:42138836–42192730	53,894	3.47E−03	0.01	*hsa-mir-4310, SPTBN5, MIR4310*
Deletion Peak 49	2	16q12.2	chr16:53968071–55360473	1,392,402	0.01	0.01	*IRX5, IRX3, CRNDE*
Deletion Peak 54	8	18q21.1	chr18:43604458–43796648	192,190	0.04	0.04	*ATP5A1, HAUS1, PSTPIP2*
Deletion Peak 55	10	18q23	chr18:77246915–77733812	486,897	2.64E−04	2.84E−04	*CTDP1, KCNG2, PQLC1, HSBP1L1, NFATC1*
Deletion Peak 66	125	22q11.22	chr22:23162356–23404171	2.42E+05	2.71E−277	5.01E−277	*IGL locus*

The most frequently amplified regions were found in 11p13 (adjacent to the *WT1* locus), 8q24.13 (*FAM91A1*), 3q25.1 (*PFN2* and *RNF13*), 18q22.1 (*TMX3*), 3q29 (*ACAP2*), 17q11.2 (*SUZ12*), 14q32.33 (adjacent to the *AKT1* locus), 13q13.3 (*NBEA/BCL8B*), 1q25.2 (*RFWD2*), 5q35.3 (*FLT4*), 19p13.3 (adjacent to the *APC2* locus), 1p22.2 (*HFM1*), and 1p31.1. Similarly, the most recurrent focal deletions were detected in 13q14.2 (*DLEU1* locus), 11q22.3 (*ATM* locus), 14q21.1 (*MIA2*), 11q14.3 (*NOX4*), 14q32.33 (*IGH* locus), and 4q13.2 (*UGT2B15* and *UGT2B17* loci). Moreover, we observed frequent deletions of immunoglobulin loci and recurrent amplified regions at TCR genes, likely reflecting deletions present in the T lymphocytes within control samples.

Three focal deletions were associated with TTT (BH *q*-value < 0.05): 11q22.3 (*ATM* locus), 14q32.33 (*IGH* locus), and 7q21.2 (*CDK6* locus). 11q22.3 loss was associated with shorter survival too. No event was associated with TTT or OS after adjusting for *IGHV* status, sex, and disease stage (BH *q*-value < 0.05). No recurrent gain was associated with treatment-free survival or OS. The association results can be consulted in **Supplementary Table 1**.

Furthermore, *SETD2* deletions (nine cases) and *IRF4* gains (five cases) were nearly significant (GISTIC residual *q*-values of 0.08 and 0.06, respectively) and associated with clinical evolution. *SETD2* deletion was associated with shorter treatment free survival (*p*-value 1.3 × 10^−8^) independent of *IGHV* status, sex, and disease stage (*p*-value 2.3 × 10^−3^); and it was also associated with shorter survival (*p*-value 9.9 × 10^−3^) but not independently of *IGHV* status (*p*-value 0.24). Amplifications in *IRF4* were associated with shorter survival (*p*-value 6.4 × 10^−5^) in a partially *IGHV*-independent manner (*p*-value 0.025). Nevertheless, this finding must be interpreted with caution due to the position of *IRF4* near the telomeric end of the short arm of chromosome 6.

### Correlation of Focal CNAs With Gene Expression

We detected significant correlations between some recurrent CNAs and their correspondingly encoded genes (**Supplementary Table 2**). As expected, deletions in 11q22.3 and 13q14.2 and the expression of their respective genes (*ATM, FDX1*, and *RDX* in the first case, and *DLEU1, DLEU2*, and *KCNRG* in the second case). Deletions in 6q21 were correlated with lower expression of *CDK19*, and so did those in 14q21.1 with the expression of *MIA2*. Furthermore, we detected significant correlations between amplifications in 3q25.1 and 3q29 and expression of their target genes *PFN2* and *ACAP2*, respectively. Surprisingly, an inverse correlation was observed between 11q14.3 deletions and *FOLH1B* expression.

Similarly, we detected significant correlations between 19 of these CNAs and the expression of 926 protein-coding genes (*q*-value < 0.01; **Supplementary Table 3**). The CNAs with more correlated genes were deletions in 13q14.2 (389 genes), 12q24.33 (*ULK1* locus, 170 genes), 6q21 (*CDK19* locus, 79 genes), 11q22.3 (*ATM* locus, 39 genes), and 3q25.33 (*IL12A* locus, 26 genes), as well as amplifications in 18q22.1 involving *TMX3* (135 genes).

Nonetheless, this study is probably underpowered to detect CNA–transcript correlations due to the fact that only 50% of the exome-seq samples had matched RNAseq data of purified CLL cells.

### Broad CNA Regions and Their Association With Clinical Evolution

Recurrent broad amplifications and deletions were detected in the CLL genome (**Table 3**, **Supplementary Figure 1**). Among the amplifications, the most frequent were found in chromosomes 12 (60 cases, 13.6% of patients), 2p (10 patients), 8q (6 patients), 18q (6 patients), 18p (5 patients), and 3q (5 patients). Similarly, the most recurrent large deletions were detected in 17p (8 patients), 18p (7 patients), and 8p (6 patients).

**Table 3:** Significantly enriched broad cytogenetic aberrations in the chronic lymphocytic leukemia (CLL) genome.

Arm	Genes	Amp frequency	Amp frequency score	Amp z-score	Amp q-value	Del frequency	Del frequency score	Del z-score	Del q-value
1p	2,121	0	0	−0.613	0.92	0	0	−0.613	0.93
1q	1,955	0	0	−0.729	0.92	0	0	−0.729	0.93
2p	924	0.02	0.02	6.99	1.81E−11	0	0	0.446	0.93
2q	1,556	0	0	1.16	0.48	0	0	0.106	0.93
3p	1,062	0	0	0.543	0.83	0	0	−1.17	0.93
3q	1,139	0.01	0.01	3.26	3.59E−03	0	0	−1.13	0.93
4p	489	0	0	−1.38	0.92	0	0	0.0708	0.93
4q	1,049	0	0	0.53	0.83	0	0	−1.17	0.93
5p	270	0	0	−0.0742	0.92	0	0	−1.45	0.93
5q	1,427	0	0	0.965	0.59	0	0	−1.01	0.93
6p	1,173	0	0	−1.13	0.92	0	0	−1.13	0.93
6q	839	0	0	−1.25	0.92	0.01	0.01	1.14	0.67
7p	641	0	0	−1.33	0.92	0	0	−0.574	0.93
7q	1,277	0	0	−0.151	0.92	0	0	−0.151	0.93
8p	580	0	0	0.155	0.92	0.01	0.01	3.12	0.01
8q	859	0.01	0.01	3.58	1.36E−03	0	0	−0.433	0.93
9p	422	0	0	−1.4	0.92	0.01	0.01	0.74	0.93
9q	1,113	0	0	−1.15	0.92	0	0	0.594	0.93
10p	409	0	0	−1.41	0.92	0	0	−1.41	0.93
10q	1,268	0	0	−1.08	0.92	0	0	−1.08	0.93
11p	862	0	0	−0.442	0.92	0	0	−0.442	0.93
11q	1,515	0	0	−0.969	0.92	0	0	1.09	0.67
12p	575	0.14	0.14	43.2	0	0	0	−1.26	0.93
12q	1,447	0.14	0.14	58.9	0	0	0	−0.933	0.93
13q	654	0	0	−1.32	0.92	0	0	−1.32	0.93
14q	1,341	0	0	−1.05	0.92	0	0	−0.1	0.93
15q	1,355	0	0	−0.0881	0.92	0	0	−1.05	0.93
16p	872	0	0	−1.24	0.92	0	0	−0.439	0.93
16q	702	0	0	−1.31	0.92	0	0	−0.54	0.93
17p	683	0	0	−1.3	0.92	0.02	0.02	4.8	3.16E−05
17q	1,592	0	0	0.144	0.92	0	0	1.22	0.67
18p	143	0.01	0.01	1.9	0.12	0.02	0.02	3.24	0.01
18q	446	0.01	0.01	2.92	9.84E−03	0	0	−1.39	0.93
19p	995	0.01	0.01	2.16	0.07	0	0	0.492	0.93
19q	1,709	0.01	0.01	3.73	9.33E−04	0	0	1.44	0.64
20p	355	0	0	−0.714	0.92	0.01	0.01	1.39	0.64
20q	753	0	0	−0.51	0.92	0	0	−1.29	0.93
21q	509	0.01	0.01	0.823	0.67	0	0	0.0945	0.93
22q	921	0	0	−1.22	0.92	0	0	0.411	0.93

**Figure 3:**
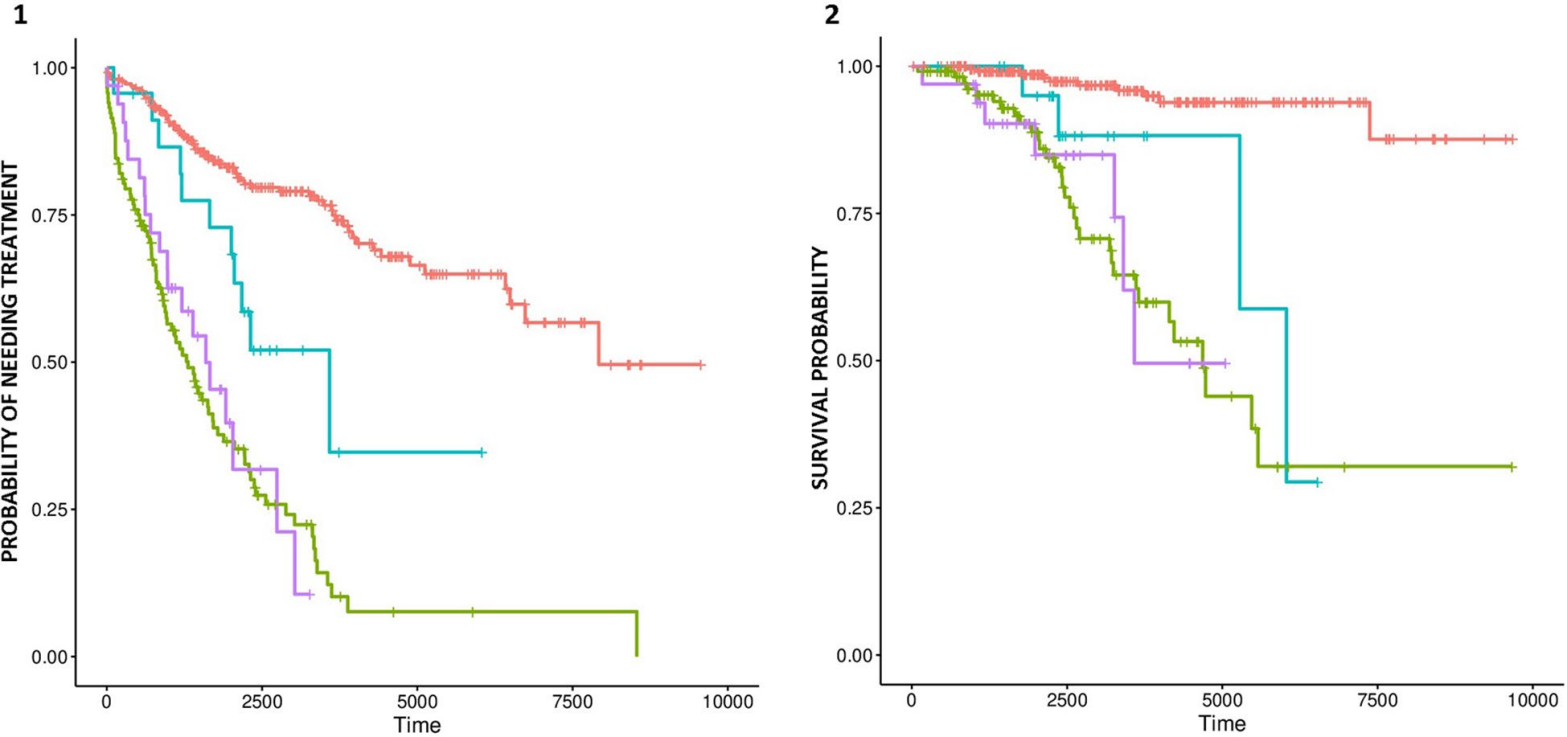
Probability of treatment need **(A)** and survival **(B)** for trisomy 12-negative patients with mutated *IGHV* (red line), trisomy 12-positive patients with mutated *IGHV* (blue line), trisomy 12-negative patients with unmutated *IGHV* (green line), and trisomy 12-positive patients with unmutated *IGHV* patients (purple line).

Amplifications in chromosomes 12, 2p, and 8q, as well as deletion of 17p, were significantly associated with shorter TTT (**Supplementary Tables 4** and **5**). Furthermore, deletion of 17p was significantly associated with TTT independently of *IGHV* status, sex, and Binet staging. Amplifications in chromosomes 12 and 8q were associated with shorter OS, but no event was significant after adjusting for *IGHV* status, age, and Binet staging. Nonetheless, we detected a significant difference in OS and TTT between *IGHV*-mutated cases with and with and without trisomy 12 (**Figures 3A, B** respectively).

### Regions of CNN-LOH

Control-FREEC identified 63 regions of CNN-LOH with a genotype uncertainty below 5% (**Supplementary Table 6**). Ten events were located at 1p, six of which affected *ARID1A*. By comparing with mutation data published by Puente et al. (2015), none of these samples bore concurrent non-synonymous mutations in *ARID1A*. Other three events were detected at 1q, with a minimally affected region on 1q21.3, which holds likely driver genes such as *PI4KB* and *IL6R/CD126*. Four CNN-LOH events with a minimally involved region in 9q34.13–q34.3 involved the *NOTCH1* gene. One of these cases also had a frameshift deletion in *NOTCH1*. Three events at 11p15.5–15.4 affected the imprinted locus of *IGF2* and *CDKN1C*. Eight CNN-LOH events affected the 11p11.2–q13.2 region. Two different samples had events at 11q involving the *ATM* locus, both of which also had non-synonymous *ATM* mutations. Three events were located in 16p13.3–p13.11, which involve the *CREBBP* gene. None of these had mutations in *CREBBP*. Ten CNN-LOH events affected chromosome 17, three of which included the *TP53* locus. Among the latter, two had non-synonymous mutations in *TP53*. Four samples had CNN-LOH events at chromosome 20, all of which affect the *ASXL1* gene but without any concurrent mutation in it. It is interesting to mention that only two of the CNN-LOH events overlapped with those reported by Puente et al. (2015) using SNP array technology.

## Discussion

The detection of new cytogenetic aberrations using targeted sequencing takes advantage of the increased sensitivity of this technique in order to detect small events that would be otherwise difficult to identify using array-based techniques. CLL is characterized by large-scale cytogenetic alterations (Döhner et al., 2000), but focal rearrangements have been studied to a lower extent. Using sequencing data originally produced by Puente et al. (2015), here, we report the existence of 54 putatively recurrent focal CNAs in the CLL genome.

Recurrent focal amplifications were shorter than deletions, mostly involving one or two genes. The most significantly enriched focal gains affected the loci of *SUZ12, WT1, HFM1, RFWD2, FLT4*, and *TTNI3K*. On the contrary, focal deletions were wider and tended to span more than two genes. As expected, among the most significant deletions were those in 13q14.2 and in 11q22.3. Nevertheless, other highly significant regions involved the loci of genes such as *NOX4*, a component of the NADPH oxidase complex (Guo and Chen, 2015), and *MIA2*, a tumor suppressor gene (Hellerbrand et al., 2008). Deletions in *SETD2*, 11q22.3, and 14q32.33 were associated with shorter time to first treatment, and gains of *IRF4* were independently associated with short survival. Furthermore, we could detect significant positive correlations between six recurrent CNAs and the expression of genes encoded in their respective loci, as well as correlations between 19 CNAs and the expression 926 protein-coding genes genome wide.

Focal recurrent gains and losses tended to target genes that participate in oncogenic pathways. For example, five amplified genes (*HFM1, ANAPC10, TAF5, COBRA1*, and *SYCE3*) and one deleted gene (*FAM175A/ABRAXAS*) are involved in DNA transcription, replication, and repair mechanisms. Both *COBRA1* and *FAM175A* physically interact with the tumor suppressor *BRCA1* (Castillo et al., 2014; Yun et al., 2018), whereas *ANAPC10* belongs to the anaphase-promoting complex/cyclosome family of proteins that control sister chromatid segregation and cytokinesis (Chang et al., 2014). The amplified genes *PHF21A*, *PCGF6*, and *SUZ12* encode epigenetic regulators with repressor activity (Iwase et al., 2006; Vizán et al., 2015; Zhao et al., 2017). Other genes targeted by recurrent events participate in important pathways. This is the case of the amplified oncogenes *AKT1* (Hyman et al., 2017) and *WT1* (Bergmann et al., 1997), and of the receptor tyrosine kinase gene *FLT4*, which regulates lymphangiogenesis and tumor metastasization to lymphatic vessels (Lee et al., 2016). Likewise, the deleted genes *AMD1, TP53INP1, ULK1*, and *NFATC1* are also important in tumorigenesis. *AMD1* and *TP53INP1* participate in metabolic pathways, and both have tumor suppressor activity (Scuoppo et al., 2012; Saadi et al., 2015), whereas *ULK1* plays a decisive role in autophagy initiation (Zachari and Ganley, 2017) and *NFATC1* maintains an anergic phenotype in CLL cells (Märklin et al., 2017). Finally, two cyclin-dependent kinase genes were recurrently deleted: *CDK6* and *CDK19*. Deletions in *CDK6* were associated with shorter time to first treatment, whereas those in *CDK19* were correlated with a reduced expression of the gene. *CDK19* is a component of the mediator kinase module, which associates with the mediator complex in order to regulate diverse cellular functions (Dannappel et al., 2019), and *CDK6* is a promoter of cell-cycle progression (Kollmann and Sexl, 2013). The role of both genes in the pathogenesis of CLL needs further clarification.

Finally, recurrent broad cytogenetic aberrations characteristic of CLL were identified at the expected frequency, as in the case of trisomy 12, 17p deletion, amplification of 8q, and loss of 8p (Blanco et al., 2016). Interestingly, we detected a significant adverse time to event and OS effect of trisomy 12 among *IGHV*-mutated cases. The importance of *IGHV* mutation as a predictor of disease evolution within the group of patients with trisomy 12 was previously reported by others (Bulian et al., 2017; Roos-Weil et al., 2018), but to our knowledge, this is the first report about the prognostic importance of trisomy 12 among *IGHV*-mutated cases. Furthermore, new CNN-LOH events affecting CLL drivers were also detected, most of which had not been described in previous array-based analysis of the CLL genome (Novak et al., 2002; Pfeifer et al., 2007). These CNN-LOH events affected the *ATM, NOTCH1, TP53, ARID1A, ASXL1, CREBBP*, and *PI4KB/IL6R* loci, as well as the telomeric region of 11p. Only part of these mutations had concurrent mutations in their corresponding driver genes, suggesting the existence of other mechanisms of pathogenicity.

Detection of copy number changes based on exome-sequencing has been proven to be prone to false positives by some studies (Rieber et al., 2017). In this analysis, we have included patient matched control samples, we applied stringent thresholds in order to minimize false detections, and we recapitulated most of the cytogenetic findings of CLL in the expected frequency. Furthermore, we could correlate the presence of this structural aberrations with changes in gene expression in a subgroup of patients, although we believe that this study may be underpowered to detect such associations.

In conclusion, our study presents proof-of-concept evidence for the existence of new focal recurrent CNAs and CNN-LOH in the genome of CLL, some of which influence clinical outcome. Furthermore, we observed that some of these novel events have significant correlations with gene expression changes. The results are concordant with the possible involvement of a set of oncogenes and tumor suppressors in the development of CLL. These results should be considered a “proof of concept,” and their existence and functionality should be validated in the future.

## Author Contributions

AM, BA, and JB designed the study. AM performed the research and analyzed the data. AM, BA, JD, MG and JB analyzed the results and wrote the paper.

## Funding

This research has been performed without funding. The publication costs associated with this manuscript have been partially paid by Roche Pharmaceuticals. The funder played no role in the study design, data collection, analysis, results interpretation, writing or in the decision to submit this paper for publication.

## Conflict of Interest Statement

The authors declare that the research was conducted in the absence of any commercial or financial relationships that could be construed as a potential conflict of interest.

## Supplementary Material

Supplemental Figure 1 Heatmap representation of genomic profiles made using segmented copy number data of the CLL genomes.

Supplementary Table 1 Association of focal amplifications and deletions with TTT and OS. Only those events affecting at least 5 patients of this cohort were selected for the analysis.

Supplementary Table 2 Significant correlations detected between CNA events and expression of genes encoded in their respective loci.

Supplementary Table 3 Significant correlations detected between CNA events and any protein-coding genes genome-wide (q-value <0.01).

Supplementary Table 4 Association of large-scale deletions with TTT and OS. Only those events affecting at least 5 patients of this cohort were selected for the analysis.

Supplementary Table 5 Association of large-scale amplifications with TTT and OS. Only those events affecting at least 5 patients of this cohort were selected for the analysis.

Supplementary Table 6 Summary of the detected LOH events detected, along with candidate gene annotation and its mutation status in the data published by *Puente et al*.
