# Supplementary figures and images for: New Recurrent Structural Aberrations in the Genome of Chronic Lymphocytic Leukemia Based on Exome-Sequencing Data
Source: Front Genet. 2019 Sep 20;10:854. doi: 10.3389/fgene.2019.00854 (PMC6764480; doi:10.3389/fgene.2019.00854)

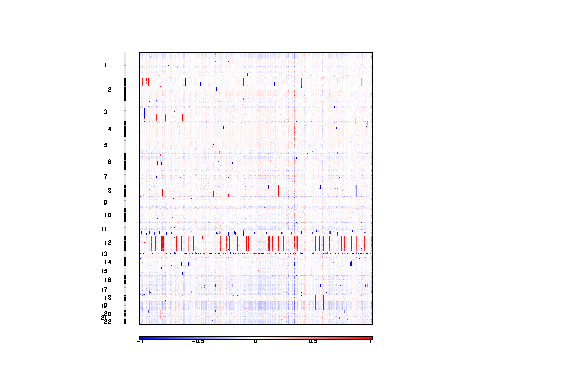

Supplement: Supplemental Figure 1 — Heatmap representation of genomic profiles made using segmented copy number data of the CLL genomes. [file DataSheet_1.zip › Supplementary Figure1.png]
